# Altered circulating levels of B cell growth factors and their modulation upon anti-tuberculosis treatment in pulmonary tuberculosis and tuberculous lymphadenitis

**DOI:** 10.1371/journal.pone.0207404

**Published:** 2018-11-14

**Authors:** Gokul Raj Kathamuthu, Kadar Moideen, Vaithilingam V. Banurekha, Dina Nair, R. Sridhar, Dhanaraj Baskaran, Subash Babu

**Affiliations:** 1 National Institutes of Health-NIRT-International Center for Excellence in Research, Chennai, India; 2 National Institute for Research in Tuberculosis (NIRT), Chennai, India; 3 Government Stanley Medical Hospital, Chennai, India; 4 Laboratory of Parasitic Diseases, National Institute of Allergy and Infectious Diseases, National Institutes of Health, Bethesda, Maryland, United States of America; National Institute for Infectious Diseases (L. Spallanzani), ITALY

## Abstract

B cell activating factor/a proliferation-inducing ligand (BAFF/APRIL) are members of the tumor necrosis factor alpha (TNF) α family of ligands, which are essential for B cell survival, development, and modulation of the immune system. To examine the association of circulating levels of BAFF and APRIL with pulmonary tuberculosis (PTB) and tuberculous lymphadenitis (TBL), we measured the systemic levels of APRIL and BAFF in individuals with PTB, TBL, latent tuberculosis (LTB) and healthy controls (HC). Further, we also examined the pre and post-treatment plasma levels of above-mentioned parameters in PTB and TBL individuals upon completion of anti-TB chemotherapy. Next, the association of these cytokines either with extent of disease, disease severity, bacterial burden in PTB and lymph node culture grade or the lymph node size in TBL was also assessed. Finally, ROC analysis was performed to examine the discrimination capacity of APRIL and BAFF between PTB or TBL with LTB. Our study revealed significantly diminished plasma levels of APRIL in PTB and higher plasma levels of BAFF in both PTB and TBL individuals compared to LTB and HC. Furthermore, we observed a significant increase in APRIL levels in TBL and significantly decreased plasma levels of BAFF in both PTB and TBL after the completion of successful anti-TB treatment. There was no statistically positive relationship between BAFF and APRIL levels and the extent of disease, disease severity and bacterial burden in PTB. In TBL, there was a significant correlation between APRIL (but not BAFF) levels with lymph node culture grades. In contrast, APRIL in PTB and BAFF in TBL were able to clearly discriminate from LTB in ROC analysis. In summary, our results showed altered levels of BAFF/APRIL and their modulation upon chemotherapy, suggesting that these cytokines might be involved in the immune-modulation of TB infection.

## Introduction

Despite major efforts, *Mycobacterium tuberculosis* (MTb) disease still remains a profound global burden with 6.4 million new cases, causing 10.0 million disease illness and 1.3 million deaths worldwide in 2017 [1]. It has been proposed that adaptive immunity, chiefly, antigen-specific CD4^+^T cell responses (mainly interferon (IFN) γ) plays a vital function in the outcome of MTb infection [2, 3]. Likewise, the other type 1 cytokine, tumor necrosis factor (TNF) α can also act as a defensive factor against MTb infection through the maintenance of the granuloma [4]. Exposure to Mtb typically results in latent TB infection (LTB) i.e without the active disease [5]. It is only about 5–10% of LTB that actually develops to active pulmonary tuberculosis (PTB) disease. Similarly, the most common manifestation of extra-pulmonary TB is defined as tuberculous lymphadenitis (TBL), which either disseminates through the hematogenous or via lymphatic channel [6].

Based on the reported evidence, it is clear that T-cell-mediated immunity is essential in providing protection to the host against MTb infection. However, the recent data suggest that B cells and humoral immunity can also play a significant role in resistance to MTb infection or disease [7–9]. B cells are known to play an important role in response to MTb antigens and in the maintenance of MTb granulomas in animal models [10, 11]. Similarly, modulation in the gene expression profile of TB patients during anti-TB treatment reveals alterations in gene pathways involved in humoral mediated immunity [12]. Besides, active TB disease is associated with altered levels of B cell growth factors, a proliferation-inducing ligand (APRIL, TNFSF 13a) and B cell activating factor (BAFF, TNFSF 13b). These factors belong to the TNF family, which are essential for antibody production and survival of peripheral B cells [13, 14]. Furthermore, patients with PTB are known to have an improper function of circulating B cell compartment which can be modulated upon completion of successful anti-tuberculosis treatment [15].

Since both APRIL and BAFF are crucial for B cell development and survival apart from being involved in antibody production, we sought to examine the association of these cytokines in PTB and TBL disease. Hence, we measured these B cell growth factors (BAFF and APRIL) in PTB, TBL, LTB and HC individuals using plasma samples. Our results demonstrated that diminished APRIL levels in PTB and increased BAFF levels in both PTB and TBL and their modulation upon successful chemotherapy.

## Methodology

### Study ethics

This study was approved by the Internal Ethics Committee of National Institute of Research in Tuberculosis (NIRT, NIRTIEC2010007). The written informed consent was obtained from all the study participants.

### Subjects and samples

Blood samples of active pulmonary tuberculosis (PTB, n = 44), TBL (n = 44), LTB (n = 44), and healthy control (HC, n = 44) individuals were collected from Chennai, Tamil Nadu, India between the years 2011–2017. PTB was diagnosed on the basis of smear and culture positivity for MTb. Similarly, the diagnosis of TBL was made on the basis of excision biopsy (i.e affected lymph nodes) showing positivity for bacteriology consisting of Xpert or culture positive for MTb. Lymph node size and numbers were determined by ultrasound. Disease severity in PTB was assessed by chest X-ray readings of unilateral or bilateral disease and cavitary versus no non-cavitary disease and in TBL by size and number of lymph nodes. Chest X-rays were read (in a blinded fashion) and interpreted by two independent readers. During the time of enrolment, all active PTB and TBL cases had no previous record of TB disease or administration of anti-TB treatment (ATT). All the study individuals were BCG vaccinated, HIV negative and not under any steroid treatment. Standard anti-tuberculosis treatment (ATT) was administered for 6 months to both PTB and TBL individuals. After the completion of ATT, once again the blood samples were collected from the study participants. LTB and HC were diagnosed based on the positive or negative for tuberculin skin test (TST) and QuantiFERON-TB Gold in Tube (Cellestis) ELISA, absence of X-ray or pulmonary symptoms and negative sputum smear and culture. A positive TST result was defined as an induration of at least 12mm in diameter to reduce the false positivity due to exposure of environmental mycobacteria. Only the individuals positive by both TST and QuantiFERON TB Gold were considered as LTB individuals and negative for both were considered as HC individuals.

### Plasma collection

Venous blood (10 ml) was collected in sodium heparin tubes. Plasma was separated by centrifugation at 2600 revolutions per minute (rpm) for 10 minutes at 4°C, aliquoted and stored at -80°C until further use.

### Measurement of B cell growth factors, APRIL and BAFF by ELISA

The systemic levels of APRIL and BAFF were measured by duo set ELISA (R&D Systems, Minneapolis, MN, USA). The lowest detection limits of APRIL and BAFF were 31.25 pg/ml and 15.1 pg/ml.

### Statistical analysis

Data analysis were performed using Graph-Pad PRISM Version 7.0. (GraphPad Software, Inc., San Diego, CA, USA). Geometric means (GM) were calculated using the column statistics of unpaired t test. Statistically significant differences between the groups were analysed using the Kruskal-Wallis test with Dunn’s multiple comparisons. Wilcoxon signed rank test was used to compare the pre and post-treatment cytokine levels. Mann-Whitney test was used to compare the difference in the cytokine levels with various disease parameters such as lung lesions, cavity, and involvement of lymph node and culture size. Linear trend post-test analysis was used to analyse the association between cytokine levels with sputum or lymph node culture grades (reflecting bacterial burdens). Receiver operator characteristics (ROC) curves were performed to examine the capacity of APRIL and BAFF to distinguish PTB or TBL from LTB individuals.

## Results

### Study demographics

The detailed demographics of the study population are shown in Table 1. There was a male preponderance in PTB and both PTB and TBL individuals had significantly lower weight compared to the other two groups.

**Table 1:** Demographics of the study population.

Study Demographics	PTB	LTB	HC	TBL
**No. of subjects recruited**	44	44	44	44
**Gender (Male / Female)**	30/14	22/22	25/19	21/23
**Median Age (Range)**	40 (19–54)	36 (22–65)	36 (21–58)	27 (18–51)
**Median Height, cm**	163 (147–182)	163 (146–175)	162 (143–179)	160 (140–168)
**Median Weight, kg**	45 (33–68)	59 (37–80)	61 (41–95)	45 (34–68.6)
**QuantiFERON-TB Gold**	Not done	Positive	Negative	Not done
**Tuberculin skin test, mm**	Not done	<12	<12	Not done
**Culture/smear grade (0/1+/2+/3+)**	0/12/9/23	-	-	15/27/1/1

### PTB is associated with diminished APRIL and heightened BAFF systemic levels in comparison to TBL, LTB and HC individuals

**Fig 1:**
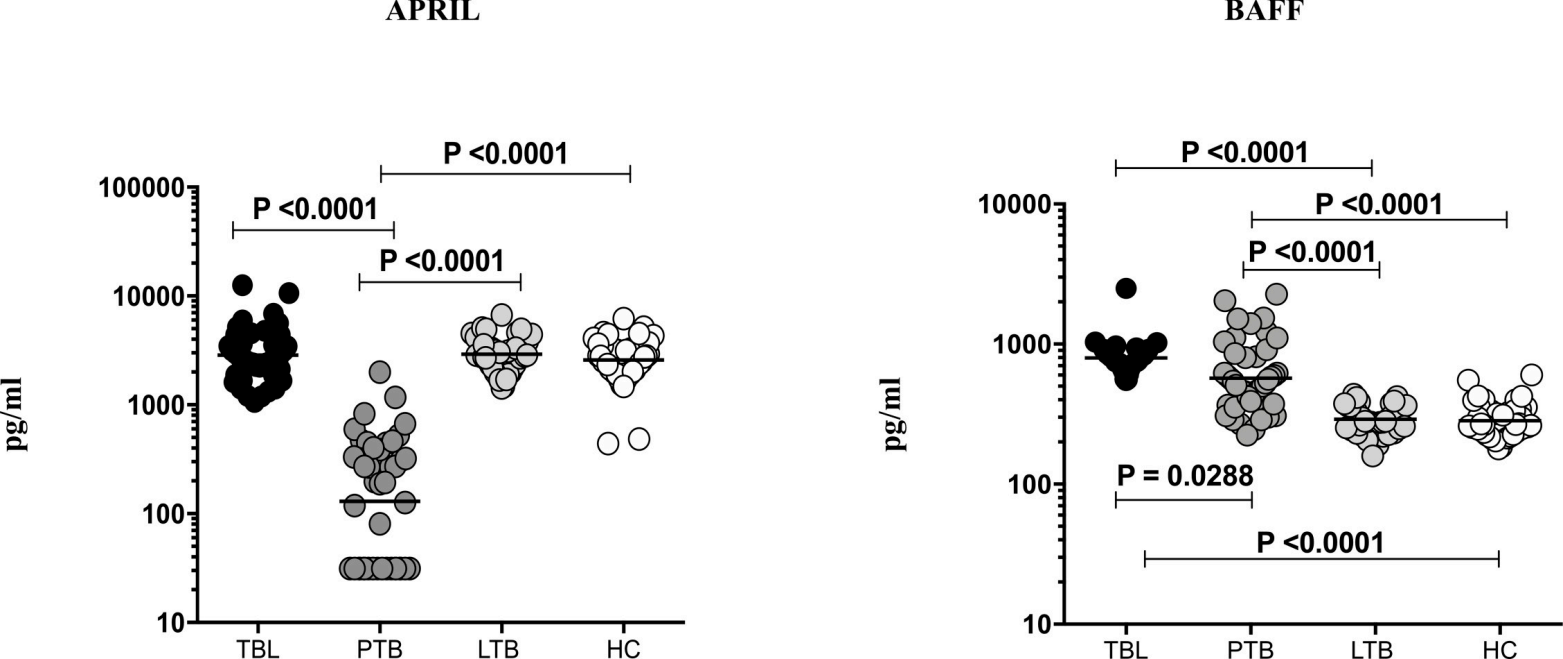
PTB and/or TBL are associated with diminished APRIL and elevated BAFF levels. The systemic levels of BAFF and APRIL were measured in active PTB (n = 44), TBL (n = 44), LTB (n = 44) and in HC (n = 44). The results are shown as scatter plots with each circle representing a single individual and the bar representing the geometric mean. P values were calculated using the Kruskal-Wallis test with Dunn’s multiple comparisons.

To determine the circulating plasma levels of B cell growth factors in TB, we measured the circulating plasma levels of APRIL and BAFF in PTB, TBL, LTB and HC individuals (Fig 1). The circulating plasma levels of APRIL was significantly diminished in PTB when compared to TBL (geometric mean (GM) of PTB is 130.1 pg/ml versus 2859 pg/ml in TBL), LTB (GM of PTB is 130.1 pg/ml versus 2921 pg/ml in LTB and HC (GM of PTB is 130.1 pg/ml versus 2582 pg/ml in HC) individuals. Similarly, the circulating plasma levels of BAFF was significantly elevated in TBL and PTB (GM of TBL is 795.9 pg/ml versus 571.7 pg/ml in PTB), when compared to LTB (GM of TBL is 795.9 pg/ml versus 290.7 pg/ml in LTB) and HC (GM of TBL is 795.9 pg/ml versus 283.2 pg/ml in HC) individuals. Hence, we demonstrate that diminished APRIL and increased BAFF plasma levels were associated with PTB.

### PTB and TBL is associated with diminished circulating plasma levels of BAFF cytokine following successful ATT

**Fig 2:**
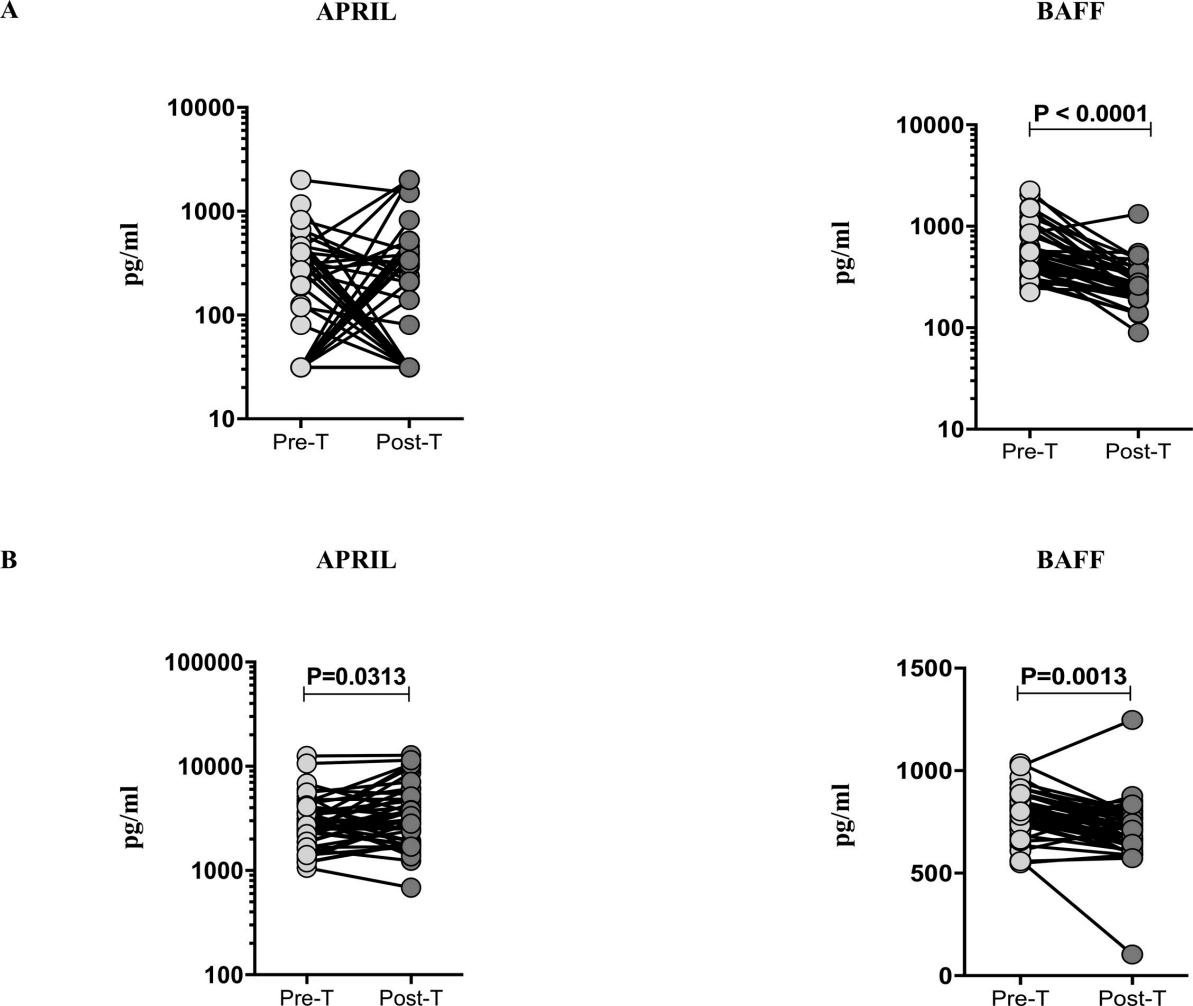
Anti-TB treatment reverses the circulating levels of BAFF in PTB and both APRIL and BAFF in TBL. The plasma levels of APRIL and BAFF (A) PTB (B) TBL were measured in the before (pre-T) and 6 months after (post-T) anti-TB chemotherapy. The data are shown as line diagrams with each line representing a single individual. P values were calculated using the Wilcoxon signed rank test.

We measured the pre and post-treatment circulating plasma levels of APRIL and BAFF in both PTB and TBL individuals (Fig 2). As shown in Fig 2A, the systemic levels of BAFF in PTB (GM of PTB pre-T is 571.7 pg/ml versus 274.5 pg/ml in post-T) was significantly diminished in post-treatment when compared to the pre-treatment levels and no significant modulation was seen in the pre and post-treatment plasma levels of APRIL (GM of PTB pre-T is 130.1 pg/ml versus 121.8 pg/ml in post-T). Similarly, we also measured the pre and post-treatment plasma levels of APRIL and BAFF in TBL individuals (Fig 2B). The plasma levels of APRIL (GM of TBL pre-T is 2859 pg/ml versus 3348 pg/ml in post-T) were significantly elevated and BAFF (GM of TBL pre-T is 795.9 pg/ml versus 690 pg/ml in post-T) plasma levels were significantly decreased in TBL individuals. Thus, successful ATT of PTB and TBL results in significantly diminished plasma levels of BAFF.

### No relationship between APRIL and BAFF cytokine levels with the extent of disease, disease severity and bacterial burden in PTB

**Fig 3:**
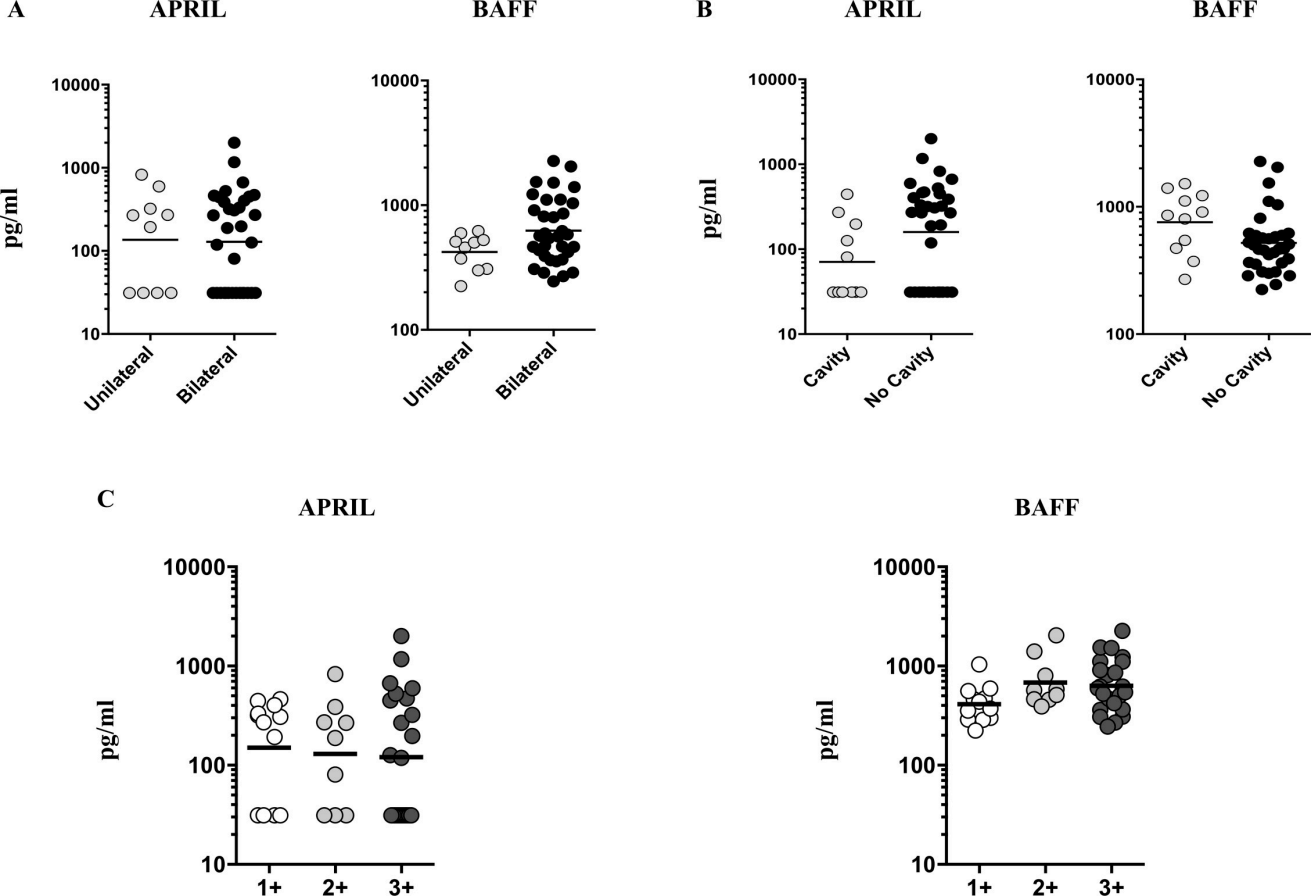
No association of circulating levels of B cell growth factors with extent of disease, disease severity or bacterial burdens among PTB individuals. (A) The plasma levels of B cell growth factors (APRIL and BAFF) in PTB individuals with unilateral versus bilateral, reflecting the extent of disease. (B) The plasma levels of B cell growth factors in PTB individuals with cavity versus non-cavity disease, reflecting the disease severity were shown. The data were shown as bar graphs displaying geometric means and 95% confidence intervals. P values were calculated using the Mann-Whitney test. (C) The relationship between the plasma levels of B cell growth factors and smear grades measured using sputum smears was examined in PTB individuals. The data are denoted as bar graphs showing geometric means and 95% confidence intervals. P values were calculated using linear trend post-test analysis.

To examine the association of B cell growth factors with the extent of disease, disease severity and bacterial burden in PTB, we examined the circulating plasma levels of APRIL and BAFF in PTB individuals either with unilateral versus bilateral, cavity versus no cavity and different smear grades (Fig 3). The plasma levels of APRIL and BAFF exhibited no significant difference when PTB individuals with bilateral disease were compared to unilateral disease (Fig 3A). Similarly, the circulating levels of APRIL and BAFF displayed no significant differences in PTB individuals with cavitary or without cavitary disease (Fig 3B). Finally, to determine the relationship between the APRIL and BAFF levels with bacterial burdens in PTB, we examined their systemic levels with respective smear grades which are classified as 1+, 2+ and 3+. As shown Fig 3C, the systemic levels of APRIL and BAFF did not exhibit any significant relationship with the smear grades of PTB individuals. Thus, we suggest that none of the B cell growth factors were significantly correlated with extent of disease, disease severity or bacterial burdens in PTB.

### Significant association of APRIL with culture grades of TBL

**Fig 4:**
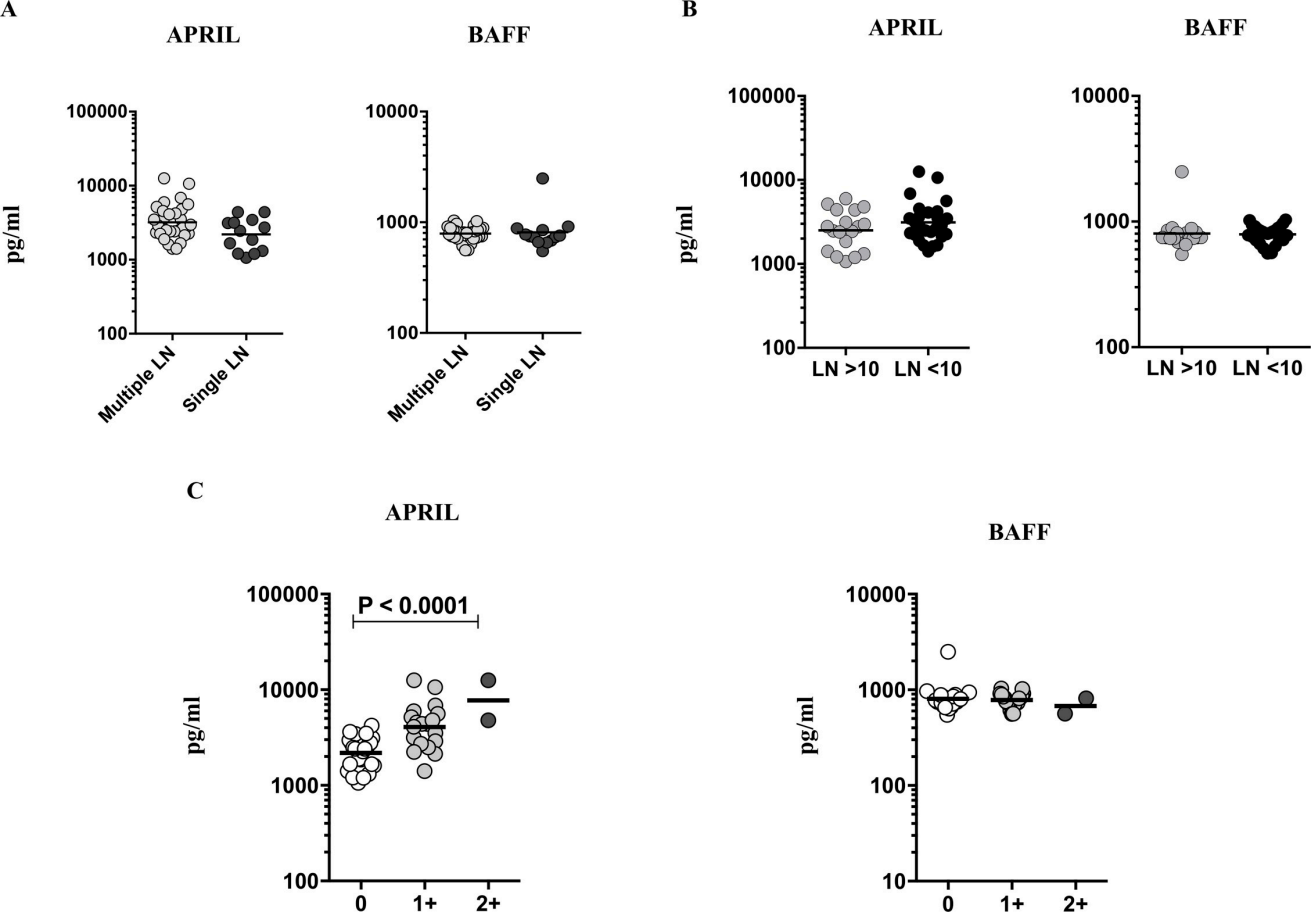
Positive association of circulating levels of B cell growth factor (APRIL) with lymph node (LN) culture grades of TBL individuals. (A) The plasma levels of B cell growth factors (APRIL and BAFF) in TBL individuals with the multiple and single LN. (B) The plasma levels of APRIL and BAFF in TBL individuals were correlated with LN size. Results were shown in scatter plots, with each circle representing a single individual and the bars indicating the geometric means. P values were calculated using the Mann-Whitney U test. (C) The relationship between the APRIL and BAFF plasma levels with LN culture grades were measured in TBL individuals. The data are denoted as scatter plots, with each circle representing a single individual and the bars indicating the geometric means. P values were calculated using linear trend post-test analysis.

To examine the association of B cell growth factors with disease extent or bacterial burdens in TBL, we measured the plasma levels of APRIL and BAFF and correlated them with their respective lymph node numbers, size or culture grades (Fig 4). As shown in Fig 4A, the systemic levels of APRIL and BAFF did not exhibit any significant difference between multiple or single lymph node involvement. Similarly, no significance was associated with the plasma levels of APRIL and BAFF upon comparison with TBL lymph node size (Fig 4B). Finally, plasma levels of APRIL were significantly associated with TBL culture grades (Fig 4C). Therefore, we demonstrate that APRIL plasma levels were significantly associated with bacterial burden and culture grades of TBL.

### ROC analysis of APRIL and BAFF cytokines

**Fig 5:**
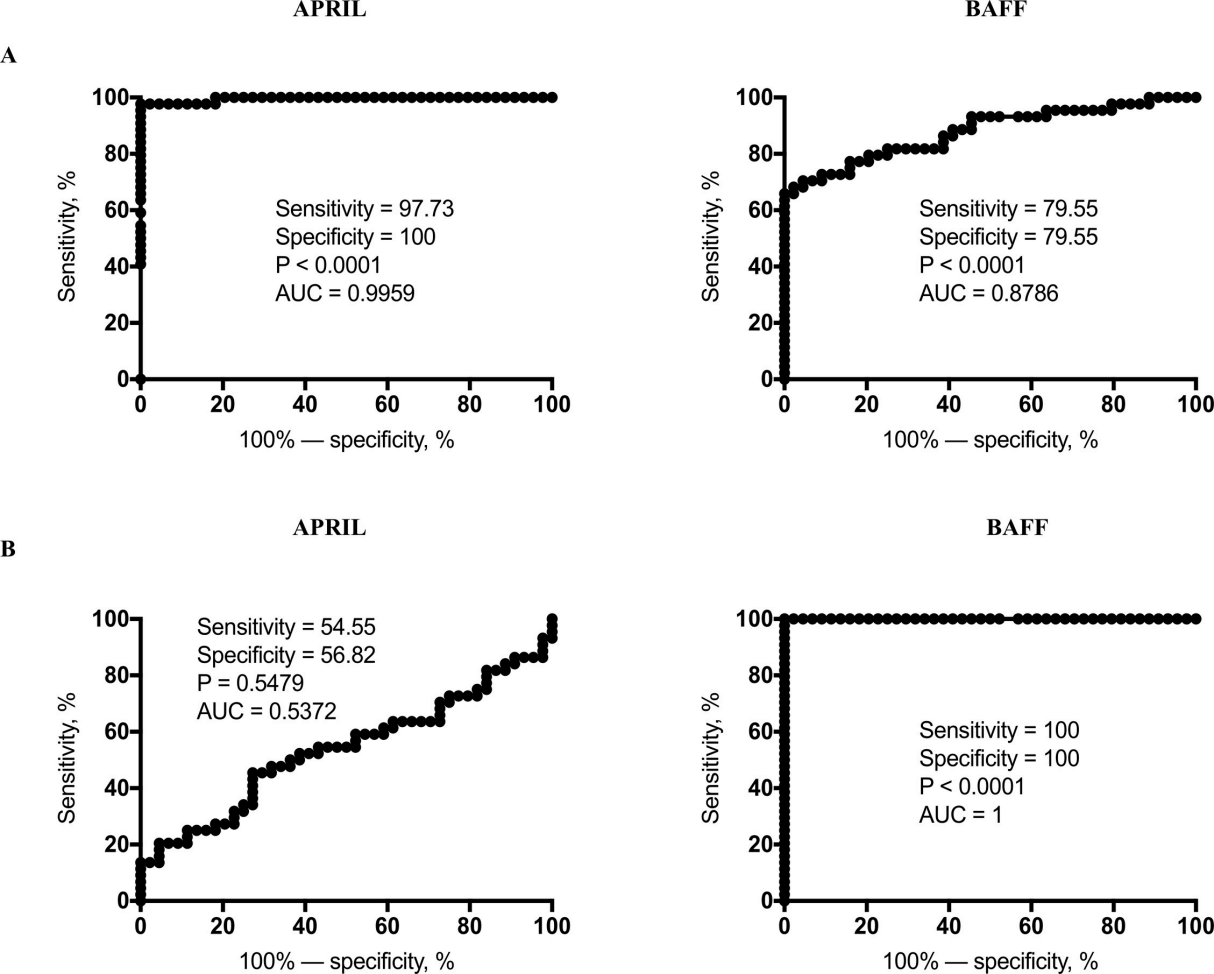
Receiver operating characteristic (ROC) analysis of APRIL and BAFF in PTB/TBL individuals. ROC analysis was performed to determine the sensitivity, specificity and area under the curve using the plasma levels of APRIL and BAFF cytokines between (A) PTB versus LTB and (B) TBL versus LTB to calculate the ability of these factors to distinguish PTB/TBL from LTB individuals.

To examine the ability of systemic (APRIL and BAFF) markers in discriminating PTB or TBL from LTB individuals, we performed ROC analysis of APRIL and BAFF cytokines using their plasma levels (Fig 5). Our analysis showed that APRIL (sensitivity-97.73, specificity-100, area under curve (AUC)-0.9959 and P<0.0001), but not BAFF (sensitivity-79.55, specificity-79.55, AUC-0.8786 and P<0.0001) in PTB exhibited significant discrimination in distinguishing PTB from LTB individuals (Fig 5A). Likewise, as shown in Fig 5B, BAFF (sensitivity-100, specificity-100, AUC-1 and P<0.0001) but not APRIL (sensitivity-54.55, specificity-56.82, AUC-0.5372 and P = 0.5479) in TBL exhibited significant discriminatory power to distinguish TBL from LTB individuals respectively.

## Discussion

The cellular immune response has been firmly recognized as the key factor of host protection, and it is noteworthy that the former alone is insufficient in providing immunity against TB infection [16]. Hence, defined immune parameters are required for host resistance which are still incompletely defined and different arms of immune activation are required for protection. Specifically, humoral mediated immune response against TB infection and disease development is thought to be also important [17, 18]. B cell producing antibodies deliver resistance through various mechanism starting from opsonization, complement activation, inflammasome activation, macrophage killing and signaling through Fc receptors [19–21]. In addition, mice deficient in B cells or humoral immunity are more susceptible to TB disease [22]. Moreover, B cells are efficient antigen presenting cells (APCs). Thereby, B cells contribute to host defense by activation of active CD4^+^ T cell immune response against TB disease [23]. Since both APRIL and BAFF have a significant impact in B cell survival and differentiation [14], we wanted to examine the systemic levels of APRIL and BAFF in both pulmonary and extra-pulmonary TB and compared them to LTB and HC individuals.

Our results showed that significantly increased plasma levels of BAFF were observed in both PTB and TBL disease. Higher BAFF plasma levels in the circulation were reported in plasma of active TB patients and pleural effusion (PE) of tuberculous pleurisy (TP) [13]. BAFF is a potent inducer of effective Th1 immune response [24] and upregulates Bcl2 molecule through BAFFR-mediated signaling [25] and promotes antibody class switching [26, 27]. In addition, increased BAFF levels may prevent the loss of peripheral CD4^+^ T cells from apoptotic cell death by activating Bcl2 expression. Based on these observations, we speculate that higher levels of BAFF suggest a potential ability to confront TB infection. While BAFF levels were diminished in TBL individuals following treatment, it did not reach the levels seen in LTB or HC individuals at baseline, suggesting that BAFF could possibly require a longer duration to restore to baseline levels.

Similarly, APRIL the other B cell growth factor has a main role in strengthening the effects of BAFF on B cells [26, 27]. Our data on APRIL show significantly diminished plasma levels in PTB individuals when compared to LTB and HC individuals. This data suggests that differential expression of APRIL levels in PTB versus TBL. While TBL levels are comparable to LTB and HC, PTB levels are not. In previous studies, elevated levels of APRIL were observed in pleural effusions and plasma. However, the soluble form of APRIL was present at the lower level in TB antigen-stimulated supernatants upon comparison with the unstimulated supernatants of TB patients [13]. The differences between this study and ours might be due to variations in the strain of the bacterium or due to differences in the host ethnic background, which needs to be studied further. Thus, patients with reduced APRIL plasma levels tend to have active disease (for example in PTB) and we speculate that since TBL is a less active disease, no reduction in plasma levels was observed. Similarly, after the completion of chemotherapy there was no significance observed in PTB individuals and increased levels were observed in TBL individuals. We do not have an explanation for the increase of APRIL levels following treatment of TBL. It is possible that it is a reflection of changes in the B cell compartment. Similarly, we speculate that the APRIL levels are not restored within the normal timeline of six months post-treatment in PTB individuals.

Finally, no association was observed for plasma levels of both B cell growth factors (BAFF and APRIL) upon correlation with either for unilateral or bilateral lung lesion, the extent of disease and culture grades of PTB as well. Unlike in PTB, upon correlation, APRIL plasma levels were significantly elevated when LN culture grades were increased and no association was observed either of these factors for involvement of single or multiple LN or LN size. Thus, while BAFF and APRIL are important in distinguishing PTB and/or TBL from LTB and HC, they are not markers of disease severity or extent or bacterial burdens. In ROC analysis, APRIL and BAFF exhibited high sensitivity and specificity in distinguishing PTB (for APRIL) and TBL (for BAFF) from LTB individuals suggesting that the importance of these markers in as potential biomarkers of disease. In summary, the present study demonstrates the presence of altered APRIL and BAFF plasma levels in PTB and TBL disease and their modulation following chemotherapy. The differences observed in the disease groups might reflect immunopathology or compromised ability to fight active TB disease.
